# Truncating and missense *BMPR2 *mutations differentially affect the severity of heritable pulmonary arterial hypertension

**DOI:** 10.1186/1465-9921-10-87

**Published:** 2009-09-28

**Authors:** Eric D Austin, John A Phillips, Joy D Cogan, Rizwan Hamid, Chang Yu, Krista C Stanton, Charles A Phillips, Lisa A Wheeler, Ivan M Robbins, John H Newman, James E Loyd

**Affiliations:** 1 Department of Pediatrics, Vanderbilt University, Medical Center, Nashville, TN, USA; 2 Department of Biostatistics, Vanderbilt University, Medical Center, Nashville, TN, USA; 3 Department of Medicine, Vanderbilt University, Medical Center, Nashville, TN, USA

## Abstract

**Background:**

Autosomal dominant inheritance of germline mutations in the bone morphogenetic protein receptor type 2 (*BMPR2*) gene are a major risk factor for pulmonary arterial hypertension (PAH). While previous studies demonstrated a difference in severity between *BMPR2 *mutation carriers and noncarriers, it is likely disease severity is not equal among *BMPR2 *mutations. We hypothesized that patients with missense *BMPR2 *mutations have more severe disease than those with truncating mutations.

**Methods:**

Testing for *BMPR2 *mutations was performed in 169 patients with PAH (125 with a family history of PAH and 44 with sporadic disease). Of the 106 patients with a detectable *BMPR2 *mutation, lymphocytes were available in 96 to functionally assess the nonsense-mediated decay pathway of RNA surveillance. Phenotypic characteristics were compared between *BMPR2 *mutation carriers and noncarriers, as well as between those carriers with a missense versus truncating mutation.

**Results:**

While there was a statistically significant difference in age at diagnosis between carriers and noncarriers, subgroup analysis revealed this to be the case only for females. Among carriers, there was no difference in age at diagnosis, death, or survival according to exonic location of the *BMPR2 *mutation. However, patients with missense mutations had statistically significant younger ages at diagnosis and death, as well as shorter survival from diagnosis to death or lung transplantation than those with truncating mutations. Consistent with this data, the majority of missense mutations were penetrant prior to age 36 years, while the majority of truncating mutations were penetrant after age 36 years.

**Conclusion:**

In this cohort, *BMPR2 *mutation carriers have more severe PAH disease than noncarriers, but this is only the case for females. Among carriers, patients with missense mutations that escape nonsense-mediated decay have more severe disease than those with truncating mutations. These findings suggest that treatment and prevention strategies directed specifically at *BMPR2 *pathway defects may need to vary according to the type of mutation.

## Introduction

Pulmonary arterial hypertension (PAH) is a devastating disease that affects people of all ages. The small pulmonary arteries are primarily affected, resulting in progressive pulmonary vascular remodeling that leads to increased pulmonary vascular resistance and right heart failure [1]. PAH may occur in a variety of clinical contexts, including as a sporadic disease known as idiopathic PAH (IPAH) and as a familial disease that typically occurs among family members who share a common genetic predisposition [2,3]. Germline mutations in the bone morphogenetic protein receptor type 2 (*BMPR2*) gene, a member of the transforming growth factor β superfamily, are found in the majority of individuals (≥ 75%) with PAH and a positive family history of the disease; in addition, *BMPR2 *mutations are found in 10-25% of cases of sporadic PAH [4-7]. *BMPR2*-associated PAH is an autosomal dominant disease with reduced penetrance. Therefore, the presence of a *BMPR2 *mutation in a patient with PAH, regardless of family history, implies that the patient has a heritable disease thus classified as heritable PAH (HPAH).

IPAH and HPAH are histopathologically indistinguishable, and historically perceived to be clinically identical [8]. Both display marked gender disparity, with ~2:1 female to male prevalence of each disease [9]. Despite their similarities, recent studies have suggested that PAH patients heterozygous for a *BMPR2 *mutation (carriers) have more severe disease. There is growing data to support this conclusion, as recent studies comparing HPAH patients with a *BMPR2 *mutation to IPAH patients showed that: (a) HPAH patients were less likely to respond to acute vasodilator testing [10,11]; (b) HPAH patients presented at a younger age and with more severe hemodynamic compromise at diagnosis [11,12]; (c) HPAH patients had a shorter time to death or lung transplantation. [12]

**Figure 1:**
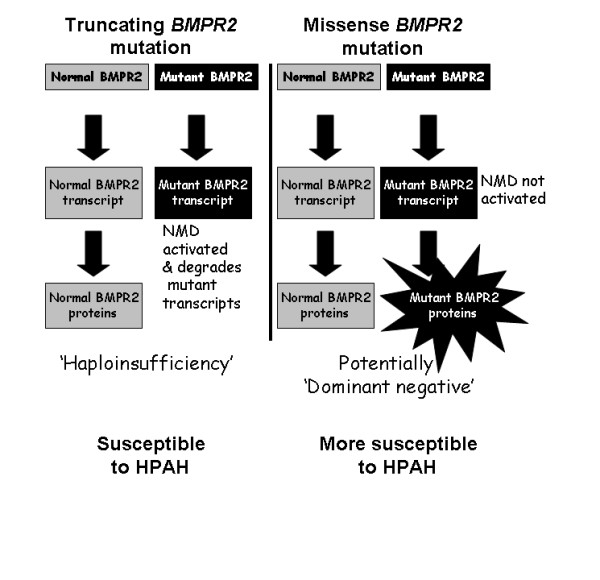
**Model of the potential impact of Nonsense-Mediated Decay (NMD) on protein expression**. Activation of the NMD pathway results in the degradation of susceptible mutant transcripts, leaving only the remaining wild-type allele that produces normal BMPR2 protein. The individual may be susceptible to disease because protein amount is quantitatively but not qualitatively reduced, resulting in 'haploinsufficiency'. Mutant RNA transcripts that are resistant to NMD may result in a mutant protein with abnormal function, including the disruption of activity by normal BMPR2 protein produced by the normal allele. The potential deleterious effect of this qualitatively but not quantitatively altered protein can result in 'dominant negative' effects, and even greater susceptibility to disease.

Heterozygosity for a *BMPR2 *mutation is neither necessary nor sufficient to cause HPAH. Disease severity among affected carriers is not uniform, and reduced penetrance (~20%) with variable age at both diagnosis and death suggest the importance of modifiers of disease expression [13]. Since different *BMPR2 *gene mutations have different affects on *BMPR2 *protein production, they are likely to cause differences in phenotype. For example, many *BMPR2 *mutations are truncating or terminating mutations that produce no functional protein product, due to mRNA degradation via activation of the nonsense-mediated decay (NMD) pathway [14]. NMD is a mechanism of RNA surveillance used by the cell to destroy RNA transcripts that would otherwise lead to the production of deleterious proteins. Thus, NMD may modify the phenotype caused by a mutation (Figure 1; see also comprehensive review by Neu-Yilik and Kulozik [14]). The expected result of a mutation whose transcript is degraded by NMD is a haploinsufficient (HI) effect due to insufficient protein production, which may cause a less severe phenotype. The vast majority, but not all, of truncating mutations activate the NMD mechanism. On the other hand, a missense mutation may result in a deleterious mutated protein product that persists because it is not destroyed by NMD [15,16].

While previous studies have demonstrated a difference in severity between *BMPR2 *mutation carriers and noncarriers, variations in severity according to *BMPR2 *mutation have not been explored. Wide variation in the severity of lung disease and survival is seen in other types of lung diseases due to mutations in a single gene, such as cystic fibrosis [17]. We hypothesized that the severity of HPAH could vary according to the type of *BMPR2 *mutation. We reasoned that carriers of a missense *BMPR2 *mutation that escapes NMD would have more severe disease than those who carry a truncating mutation. To test this hypothesis, the Vanderbilt Pulmonary Hypertension Research Group evaluated clinical data on all patients with PAH in whom genetic data were available. We found that for carriers of a truncating *BMPR2 *mutation, disease severity was milder and similar to that seen in noncarriers, while carriers of a missense mutation had more severe disease.

## Materials and methods

### Study Population

The Vanderbilt Pulmonary Hypertension Research Cohort contains clinical and biologic specimens collected over 25 years, including detailed family pedigree and medical histories of patients with HPAH and IPAH. *BMPR2 *mutations have been detected in a large proportion of subjects tested to date. They consist of nonsense or terminating mutations that are truncating, insertion-deletion mutations that lead to splicing errors and frameshift mutations (most of which are truncating), as well as missense mutations.

**Figure 2:**
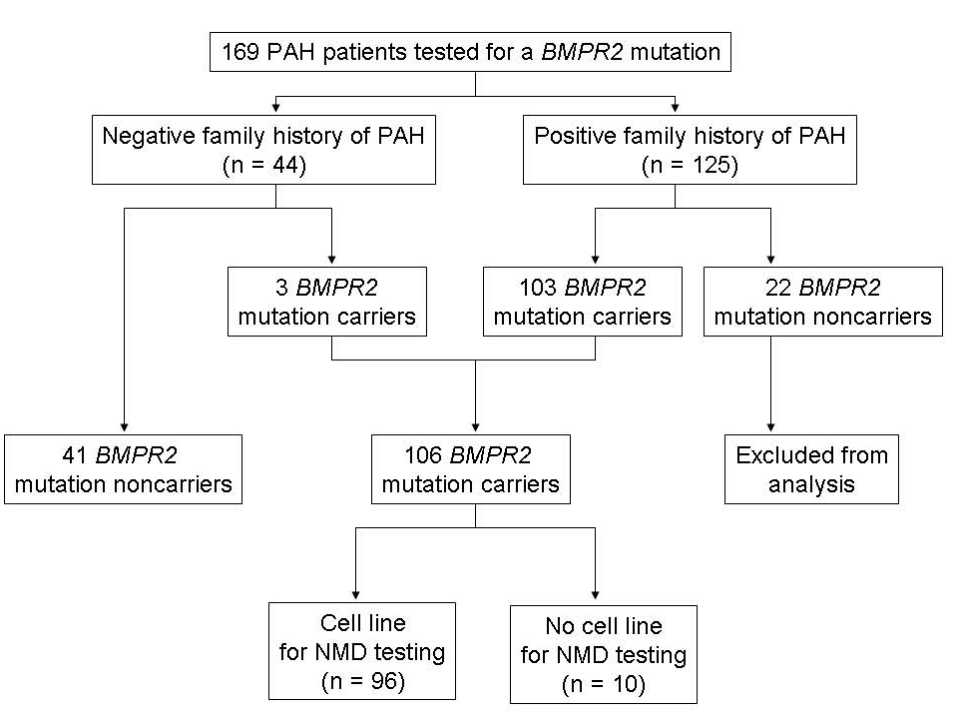
**Study subjects**. Samples from 169 consecutive subjects with PAH were tested for a *BMPR2 *mutation. Of these, 44 were from patients who had no family history. The remaining 125 were from patients who had a family history of PAH.

One hundred and sixty-nine patients had genomic DNA available (125 with a family history of PAH and 44 with no family history) for complete *BMPR2 *mutation testing. One-hundred and three of the 125 with a family history were *BMPR2 *mutation carriers (82%), while 22 were noncarriers (18%). Three of the 44 patients (7%) with no family history and presumed IPAH were *BMPR2 *mutation carriers who thus had heritable disease (HPAH). Consistent with previous studies, patients from families with multiple PAH patients with no evidence of a *BMPR2 *mutation were excluded from the analysis to decrease the risk of misclassification in the *BMPR2 *noncarrier group. Circulating lymphocytes were used to derive cell lines that were used for puromycin-based NMD studies to determine the presence or absence of NMD from 96 of the 106 *BMPR2 *mutation carriers (Figure 2).

The majority of patients (55%) were not diagnosed and treated at Vanderbilt University Medical Center (VUMC). For those patients not diagnosed and treated at VUMC, specialist physicians in their geographic regions identified HPAH patients, and our investigators reviewed all medical records for accuracy of diagnosis. We defined PAH diagnostically either by autopsy results showing plexogenic pulmonary arteriopathy in the absence of alternative causes such as congenital heart disease, or by clinical and cardiac catheterization criteria. These criteria included a mean pulmonary arterial pressure of more than 25 mm Hg with a pulmonary capillary or left atrial pressure of less than 15 mm Hg, and exclusion of other causes of pulmonary hypertension in accordance with accepted international standards of diagnostic criteria [3,18]. Clinical information concerning survival in terms of death or lung transplantation was up to date as of March 2009, the closing date for this study.

Vanderbilt Pulmonary Hypertension Research Cohort study subjects were recruited via the Vanderbilt Pulmonary Hypertension Center, the Pulmonary Hypertension Association, and the NIH Clinical Trials website (http://clinicaltrials.gov). The VUMC Institutional Review Board approved all study protocols. All participants gave informed written consent to participate in genetic and clinical studies and underwent genetic counseling in accordance with the guidelines of the American College of Chest Physicians [19]. Samples were obtained at the time of clinic visits or hospitalization or by mail via a kit for collection of whole blood and DNA.

### Genotyping and Genetic Analysis

Genomic DNA was isolated from whole blood using Puregene^® ^DNA Purification Kits (Gentra, Minneapolis, MN) according to the manufacturer's protocol. We performed *BMPR2 *gene mutation detection by sequencing exons and exon intron boundaries of genomic DNA and reverse transcriptase polymerase chain reaction (RT-PCR) analysis as previously described. [20,21] All detected *BMPR2 *mutations in this study have been previously reported, and are included in a recent summary of detectable *BMPR2 *mutations [22].

*BMPR2 *gene mutations were also assessed for NMD, using protocols previously described. [16,23] Lymphocytes from each patient were incubated with and without puromycin (250 μg/ml, Sigma-Aldrich, St. Louis, MO), which inhibits NMD, for 16 hours before harvesting. We classified mutant *BMPR2 *transcripts that were degraded in the absence of puromycin as NMD active if incubation with puromycin prevented this degradation. NMD active *BMPR2 *mutations were demonstrated to cause haploinsufficiency and classified as truncating. Mutations with transcripts not degraded in the absence or presence of puromycin were demonstrated to be NMD absent and these potentially dominant negative mutations were classified as missense [15].

### Statistical Analysis

Demographic and clinical features were compared between *BMPR2 *mutation carriers and noncarriers as appropriate with the use of χ^2 ^test or Fisher's exact test, and student's t test or Mann-Whitney U test. The primary study endpoint assessed was the continuous variable age at diagnosis. Because PAH can present at any age, and is characterized in some families by genetic anticipation, individuals affected at an earlier age and those with a shorter survival to death or lung transplantation may express a more severe form of disease [12,24].

Kaplan-Meier survival curves were used to describe differences between groups in terms of age at diagnosis, as well as survival from diagnosis to death or lung transplantation, with comparisons made using the log-rank test. Ninety-five percent confidence intervals were reported, *P *values less than 0.05 were considered statistically significant, and all tests were two-tailed. Statistical analyses were performed on a personal computer with the statistical package SPSS for Windows (Version 16.0, SPSS, Chicago). Of note, after careful analysis of the included subjects as well as the pedigrees from each family included in the study, it was concluded that the genetic relatedness of this cohort was not sufficient or appropriate to justify the use of family-based association tests that model genotypic risks.

## Results

### Clinical Characteristics, *BMPR2 *Carriers versus Noncarriers

A *BMPR2 *mutation was identified in 3 of 44 subjects (6.8%) with no family history, and 103 of 125 (82.4%) of familial cases (Figure 2). After exclusion of the 22 subjects with family history but no detectable *BMPR2 *mutation, 147 subjects were included in the analysis. This provided comparison of the 41 *BMPR2 *mutation noncarriers to the 106 carriers, who have HPAH (Table 1).

**Table 1:** Clinical characteristics of patients at the time of diagnosis of PAH.

*BMPR2 *mutation	Noncarriers (*n *= 41)	Carriers (n = 106)	P value*
Age at Diagnosis, yrs	42.0 (37.4-46.6)	36.1 (33.3-38.8)	0.04
Gender, female/male	3.1/1	2.2/1	0.43
RAP, mm Hg	12.4 (9.8-15.0)	11.1 (8.9-13.3)	0.29
Mean PAP, mm Hg	58.3 (55.0-61.5)	58.6 (55.3-61.8)	0.97
PCWP, mm Hg	10.1 (8.6-11.6)	10.6 (9.1-12.1)	0.77
CI, L/min/m^2^	1.8 (1.4-2.2)	1.9 (1.7-2.0)	0.92
PVR, mm Hg/L/min	14.0 (12.4-15.5)	18.1 (14.1-22.0)	0.08
Sv_O2_, %	56.4 (52.6-60.3)	58.4 (54.5-62.3)	0.64

* All P values calculated using Mann-Whitney U test.

† 95% Confidence Interval values calculated using univariable ANOVA

*Definition of abbreviations*: CI = cardiac index; PAH = pulmonary arterial hypertension; PCWP = pulmonary capillary wedge pressure; PAP = pulmonary artery pressure; PVR = pulmonary vascular resistance; RAP = right atrial pressure; Sv_O2 _= mixed venous oxygen saturation.

The mean age at diagnosis was significantly younger in *BMPR2 *mutation carriers (n = 106; 36.1 yrs, 95% C.I. 33.3 to 38.8) compared to noncarriers (n = 41; 42.0 yrs, 95% C.I. 37.4 to 46.6) (*P *= 0.042; Table 1). Stratification by gender revealed that these differences were only seen among females (n = 103), in whom the mean age at diagnosis was significantly younger in the *BMPR2 *mutation carriers (n = 72; 35.3 yrs, 95% CI 31.9 to 38.7), as compared to noncarriers (n = 31; 43.7 yrs, 95% C.I. 38.5 to 48.9) (*P *= 0.015; Table 1). Of note, the proportion of females with a pregnancy at the time of diagnosis or within one year of diagnosis was not different between groups (13.3% of noncarriers versus 16.7% of carriers, *P *= 0.77). While fewer males were available for analysis (n = 44), there was no difference in mean age at diagnosis (37.5 versus 36.6 years) among males.

There were no significant differences between the two groups in terms of hemodynamic characteristics at the time of diagnosis, although differences in terms of response to vasoreactivity testing were not evaluated (Table 1). Survival data were available on 138 of 147 patients studied, with 11 subjects lost to follow up. Seventy-three of the 138 patients died or underwent lung transplantation (63 *BMPR2 *mutation carriers and 10 noncarriers). Among those 73 patients, the 7 year difference in mean age at death/lung transplantation was not significantly different between carriers and noncarriers (36.6 yrs, 95% C.I. 32.9 to 40.3 versus 43.0 yrs, 95% C.I. 31.2 to 54.7; *P *= 0.389).

### Location of the *BMPR2 *mutations

*BMPR2 *gene mutations among all carriers were detected within the extracellular (n = 31), transmembrane (n = 11), kinase (n = 40), and cytoplasmic (n = 15) functional domains. One mutation was a large deletion across multiple domains, starting with the transmembrane domain. The wide distribution of mutations by location in this rare disease makes an investigation of phenotype according to location an effort that would not be expected to yield meaningful results, and a major reason that the use of nonsense-mediated decay or other means to characterize mutations phenotypically is appealing. Not surprisingly, there was no difference in age at diagnosis or death according to functional domain location of the *BMPR2 *mutations. In terms of their exonic locations, mutations were located across the *BMPR2 *gene, from exon 1 to exon 12, and no two families shared the same mutation. The wide variation in location for the various mutations made examining for phenotypic differences according to location statistically unsound.

### Clinical Characteristics, truncating versus missense *BMPR2 *mutations

**Figure 3:**
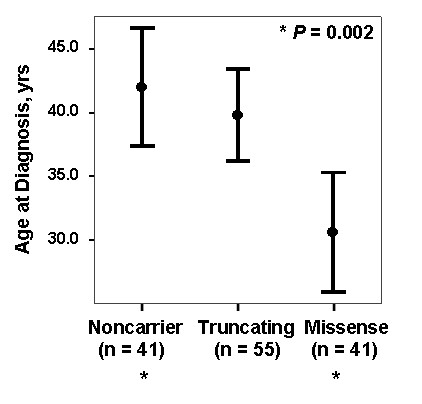
**Age at diagnosis of PAH: comparison of noncarriers, patients carrying a truncating *BMPR2 *mutation, and patients carrying a missense *BMPR2 *mutation**. Age at diagnosis is no different between noncarriers and patients carrying a truncating *BMPR2 *mutation. There is a significant difference in age at diagnosis between noncarriers and patients carrying a missense *BMPR2 *mutation (*, *P *= 0.002). Values represent mean age at diagnosis; error bars represent 95% confidence intervals.

Of the 96 *BMPR2 *mutations classified regarding NMD status as truncating or missense, 55 (57.3%) had a truncating mutation and 41 (42.7%) had a missense mutation (Table 2; Figure 2). There was no difference according to gender, with truncating mutations found in 60% of males and 50% of females (*P *= 0.38). However, there was a significant difference in mean age at diagnosis for these subjects, with PAH diagnosed in patients with truncating mutations 10 years later than in missense mutations (truncating mutations 39.9 years (95% C.I. 36.3 to 43.5) versus missense mutations 30.6 years (95% C.I. 25.9 to 35.3); *P *= 0.004). These findings also suggested that the difference in age at diagnosis between *BMPR2 *carriers and noncarriers noted in this study and by previous studies was not equal for truncating and missense mutations. In fact, while there was a substantial difference between carriers with a missense mutation and noncarriers (*P *= 0.002), there was no difference between patients with a truncating mutation and noncarriers (Figure 3). Due to limitations of sample size, comparisons between those with missense and truncating mutations were not made according to gender.

**Table 2:** Clinical characteristics of patients at the time of diagnosis of PAH, according to NMD pathway status of the *BMPR2 *mutation.

NMD status of *BMPR2 *mutation	'NMD active' (Truncating) (*n *= 55)	'NMD absent' (Missense) (*n *= 41)	P value*
Age at Diagnosis, yrs	39.9 (36.3-43.5)	30.6 (25.9-35.3)	0.004
Gender, female/male	2.6/1	1.7/1	0.82
RAP, mm Hg	11.4 (7.4-15.3)	10.5 (8.1-13.0)	0.74
Mean PAP, mm Hg	56.5 (51.6-61.5)	60.0 (54.9-65.2)	0.42
PCWP, mm Hg	11.1 (8.5-13.6)	10.3 (8.4-12.2)	0.74
CI, L/min/m^2^	1.8 (1.5-2.1)	1.9 (1.8-2.1)	0.16
PVR, mm Hg/L/min	18.4 (11.4-25.3)	17.9 (12.7-23.1)	0.78
Sv_O2_, %	58.6 (51.2-65.3)	57.3 (52.8-61.7)	0.19

* All P values calculated using Mann-Whitney U test.

† 95% Confidence Interval values calculated using univariable ANOVA

*Definition of abbreviations*: CI = cardiac index; PAH = pulmonary arterial hypertension; PCWP = pulmonary capillary wedge pressure; PAP = pulmonary artery pressure; PVR = pulmonary vascular resistance; RAP = right atrial pressure; Sv_O2 _= mixed venous oxygen saturation.

Hemodynamic parameters were not significantly different between patients with missense compared to those with truncating mutations, although differences in terms of response to vasoreactivity testing were not evaluated.

### Survival, truncating versus missense *BMPR2 *mutations

**Figure 4:**
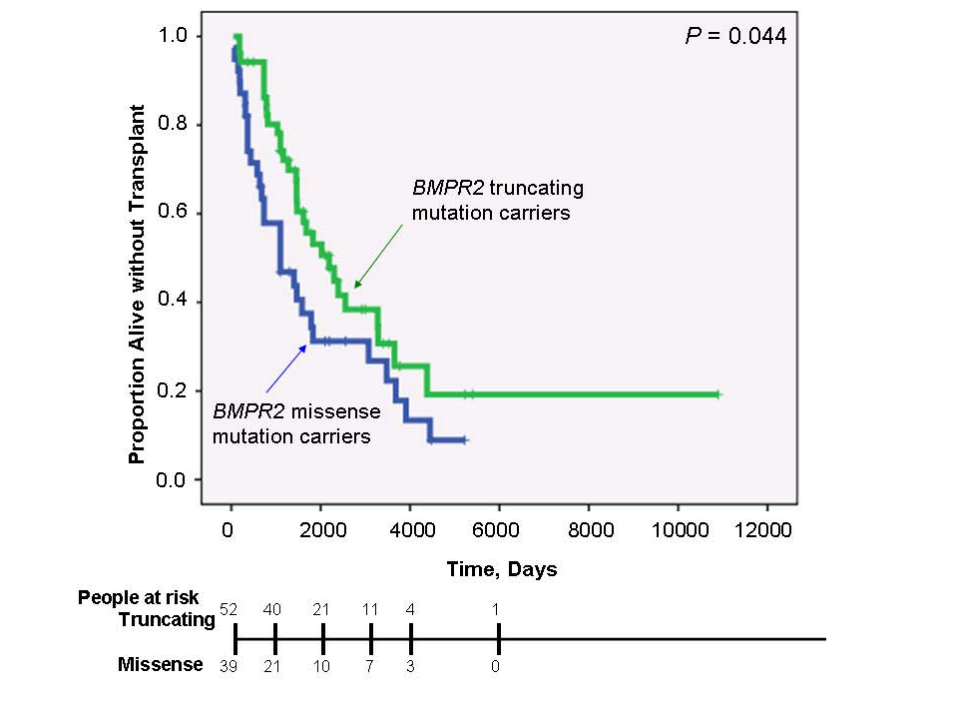
**Outcome of *BMPR2 *mutation carriers with HPAH: truncating versus missense mutation carriers**. Survival measured as time to death or lung transplantation, compared between the two groups. Survival is shorter among carriers with a missense mutation (log rank test, *P *= 0.044).

Survival data were available on 91 of the 96 patient samples analyzed for NMD pathway activation for mutation classification. Sixty-one of the 91 patients died (31 with truncating mutations and 30 with missense mutations). The use of a systemic prostanoid medication, such as epoprostenol, was not different between groups, with this class of medication used by 68.0% of patients with a truncating mutation and 82.1% of patients with a missense mutation (*P *= 0.151). Survival was different between the two groups, with a significantly shorter time to death or lung transplantation for patients with a missense mutation compared to those with a truncating mutation (1832 days, 95% CI 1272 to 2394 versus 3648 days, 95% CI 2304 to 4991; log rank test, *P *= 0.044) (Figure 4). In addition, the mean age at death/transplantation was significantly younger in the missense mutation group compared to the truncating mutation group (32.4 yrs, 95% CI 26.9 to 38.0 versus 42.0 yrs, 95% CI 37.4 to 46.6; *P *= 0.014).

### Penetrance, according to mutation type and age

**Figure 5:**
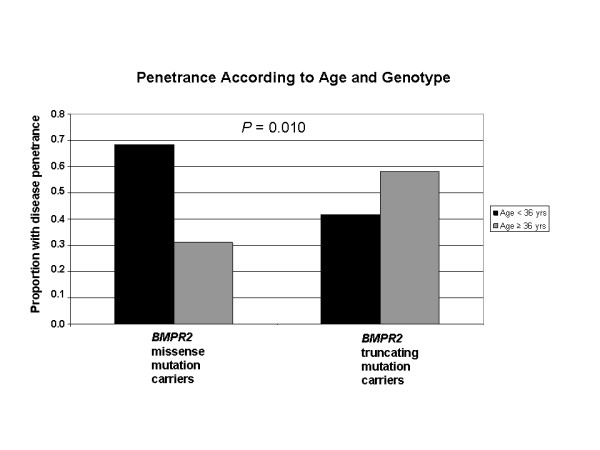
**Penetrance of PAH as a function of age**. Statistically significant difference in penetrance according to genotype and age in the comparison of the missense mutation group versus the truncating mutation group (*P *= 0.01).

An exact evaluation of penetrance among *BMPR2 *mutation carriers is currently not possible due to the inherent ascertainment bias associated with the collection of subject specimens; our cohort is enriched for patients with a *BMPR2 *mutation compared to unaffected mutation carriers. However, due to the differences in age at diagnosis and death among patients with missense and truncating mutations, we examined disease penetrance as a function of age. Due to the differences in age at diagnosis and death among patients with missense and truncating mutations, we examined disease penetrance as a function of age. The mean age at diagnosis for the entire cohort (36 years) was chosen *a priori *as a dividing point for this evaluation. We found a statistically significant difference in penetrance in the comparison of the missense mutation group versus the truncating mutation group (*P *= 0.01), with 68.3% of carriers of a missense mutation diagnosed prior to age 36 years, versus 41.3% of carriers of a truncating mutation (Figure 5). Of the 65 females within this analysis, 33 were diagnosed prior to age 36 years (18 within the missense versus 15 within the truncating mutation groups); 5 of the missense (27.8%) versus 4 of the truncating (26.7%) mutation groups were females diagnosed during or within one year of pregnancy.

## Discussion

We studied 169 patients with HPAH and IPAH with and without germline *BMPR2 *mutations, all treated at centers in the United States according to the best standard of care available. Our results support the concept that patients who are carriers of a *BMPR2 *mutation have more severe disease than noncarriers. Specifically, our results support the findings of other investigative groups that patients with a *BMPR2 *mutation presented at a younger age and had a shorter time to death or lung transplantation, while not supporting the conclusion that carriers have worse hemodynamics at the time of diagnosis [11,12]. However, we found that this is only true among females and that patients with missense mutations have more severe disease than patients with truncating mutations (Figures 1, 3, 4, 5). Significant differences in severity among those with a missense mutation are characterized by a younger age at diagnosis, younger age at death, and a shorter survival from diagnosis to death or transplant. Because these are well described indicators of disease severity, our findings suggest that in HPAH whether or not a *BMPR2 *mutation results in a stable (missense mutation) or unstable (truncating mutation) mRNA transcript is a critical modifying variable of severity [12,25,26]. As a result, therapies for treatment and prevention directed specifically at BMPR2 pathway defects may need to vary according to the type of mutation. For example, efforts are underway to identify drugs that would increase *BMPR2 *gene expression as a method of treatment and perhaps disease prevention. For carriers of an unstable mRNA transcript due to a truncating mutation, such therapies that directly increase *BMPR2 *expression might safely and effectively prevent or treat disease by increasing expression by the normal allele without changing the mutated allele's product (because it is inherently unstable). However, such a strategy could be detrimental in carriers of a mutation that produces a stable mRNA transcript associated with more severe disease, since increasing *BMPR2 *expression could increase both normal allele and mutant allele expression. The effort to tailor therapies to the type of gene mutation is consistent with efforts currently being pursued in the study of other genetic diseases, such as cystic fibrosis [27].

The functional impact of specific *BMPR2 *mutations has been incompletely investigated to date, with variable consequences for signaling activity reported [28,29]. Normally, BMPs regulate growth, differentiation, and apoptosis via an intracellular signaling cascade mediated via cytoplasmic signaling proteins known as Smads, as well as via Smad-independent pathways. The Smad family of proteins are responsible for transforming growth factor β receptor signaling to regulate gene expression [30]. There is a delicate balance of Smad signaling among the transforming growth factor β receptors, and disruption of this balance may promote a milieu amenable to the development of pulmonary vascular disease among susceptible subjects [7]. This study supports previous suggestions that truncating and missense *BMPR2 *mutations may differentially impact BMP pathway signaling, and further suggests that they do so via their differential activation of NMD [31]. NMD is a mechanism by which mRNA molecules produced by truncating mutations are detected and eliminated by the cell, resulting in no functional protein from the mutated allele. This produces a haploinsufficient effect, which magnifies the importance of the wild-type allele in protein production. Consistent with this, we recently found that the level of expression of wild-type *BMPR2 *allele is a critical factor in the pathogenesis of HPAH caused by truncating mutations [32]. In contrast, missense mutations resistant to NMD destruction may produce a mutant protein product with deleterious effects that impair or completely block the activity of the remaining normal allele. This may create a 'dominant negative effect', which affects the phenotype more severely (Figure 1) [16,22].

The comparison of a truncating versus missense mutation may be particularly important for BMP pathway signaling, which is dependent upon appropriate heterodimerization of BMPR2 with type 1 BMP receptors to activate the signaling cascade. Correct signaling requires the proper stoichiometric balance between receptors [33]. While this balance is likely perturbed by most types of *BMPR2 *mutations, it can be exaggerated by missense mutations, which cause dysfunctional heterodimers that exert dominant negative effects on BMP signaling [34]. Thus, as reported in other genetic diseases, the activation or inactivation of the NMD pathway may profoundly affect the variability of disease phenotypes in HPAH, and our findings support this concept [16,35]. From a therapeutic perspective, manipulation of the NMD surveillance pathway specific to a particular *BMPR2 *mutation might promote a less severe phenotype by changing a dominant negative effect to haploinsufficiency [36].

This study demonstrates how different variations within the same gene may influence disease, although several areas of potential criticism exist. First, because the incidence of HPAH is very low (~1 case per 1 million adults) and this study requires the creation of cell lines from each patient, like other studies of rare diseases the overall number of patients enrolled is relatively small and the collection of a replicate cohort will take many years [37]. Such an endeavor will require a multicenter collaborative effort, and the design and implementation of that investigation in an independent study population is currently underway. Second, it is possible that the 22 patients with familial PAH excluded from analysis because they did not carry a detectable *BMPR2 *mutation do carry mutations in unexplored parts of the *BMPR2 *gene (or that they have inappropriately been categorized as having a familial disease but are in fact patients with IPAH). However, our use of genomic DNA and transcript sequencing and MLPA analysis provided the most comprehensive screen for *BMPR2 *mutations described to date [20]. Third, because not every truncating genetic mutation predicted to activate the NMD pathway based upon the genotype truly activates NMD (rarely, some do not), cell line testing is required for confirmation as performed in this study. For this reason, subjects were divided for analysis according to the results of cell line testing for NMD pathway activity, not according to predicted activity (in fact, 7 of 96 subjects with cell lines tested for NMD had mutations predicted to result in activation of the NMD pathway that upon testing were not 'NMD active'). Regardless, the key issue is whether or not the mutation is capable of activating the NMD pathway or not, and the vast majority of truncating mutations do activate this pathway. Finally, the absence of significant differences in cardiovascular parameters at diagnosis in this study suggests that additional factors participate in the generation of phenotype, especially highlighting the importance of right heart adaptation to pulmonary vascular disease and the relatively small degree to which we currently understand this process at the cellular, molecular and clinical levels [38]. Efforts are underway to determine whether the response to acute vasodilator testing at the time of diagnosis is different between patients with truncating and missense mutations, as response data may predict survival. [39]

In conclusion, this study supports previous studies showing increased severity among patients with a *BMPR2 *mutation compared to those who are noncarriers, but interestingly this is only true for females and for carriers of a certain type of mutation. When we classify mutations according to if they activate (truncating mutations) or escape (missense mutations) NMD destruction of their mutant transcript we see a significant difference in severity between these groups. This supports the concept that missense mutations that escape NMD result in stable transcripts that may produce proteins that are qualitatively different. Their resultant dominant negative effects on BMP signaling make missense mutations more detrimental than truncating mutations, which cause haploinsufficiency. The haploinsufficient effects of truncating mutations occur because the total BMPR2 protein levels are quantitatively reduced to the level of expression of the single wild-type allele. These findings emphasize the need to further explore the manner in which different subclasses of *BMPR2 *mutations influence phenotype in PAH, as well as the potential role of the wild-type allele.

## Competing interests

The authors declare that they have no competing interests.

## Authors' contributions

EA provided scientific design, wrote the manuscript, performed data management, performed statistical analyses, and participated in the clinical field and laboratory work. JC, RH, CP, KS performed laboratory analyses, data management, and scientific review. LW performed clinical field work, data management and manuscript review. IR and JN contributed to project scientific oversight, and provided clinical field work and manuscript review. JP and JL had primary responsibility for scientific design, scientific oversight of this and related projects, and manuscript review.
